# Molecular docking analysis of Indole based diaza-sulphonamides with JAK-3 protein

**DOI:** 10.6026/97320630019074

**Published:** 2023-01-31

**Authors:** Manya Nautiyal, Kavitha Sekaran, Surya Sekaran, Gayathri Rengasamy, Vishnu Priya Veeraraghavan, Rajalakshmanan Eswaramoorthy

**Affiliations:** 1 Department of Biochemistry, Saveetha Dental College and Hospitals, Saveetha Institute of Medical and Technical Sciences, Saveetha University, Chennai-600077; 2 Department of Biomaterials (Green lab), Saveetha Dental College and Hospital, Saveetha Institute of Medical and Technical Science (SIMATS), Saveetha University, Chennai-600077

**Keywords:** Oxadiazoles, indole, diaza-sulphonamides, anti-cancer, JAK-3

## Abstract

JAK-3 gene is a part of an important signalling pathway in oral cancer. Therefore, it is of interest to evaluate the inhibitory properties of new indole based diaza-sulphonamides compounds against JAK3 gene. Molecular docking analysis showed that among the selected compounds (1-9), the compounds 1-4 turned out to be the most potentially capable ones to be used as ant-cancer drugs. Also, they are proved to be non-toxic.

## Background:

Heterocyclic compounds are recognized as intriguing scaffolds to incorporate in bioactive small molecules, due to the crucial role that heteroatoms cover in physiological processes. Indeed, more than 85% of biologically active compounds bear at least one heterocyclic moiety. In this context, oxadiazoles are small five-membered heterocycles, composed of two carbon, one oxygen, and two nitrogen atoms, which attracted a lot of interest in different scientific disciplines: from medicine and agrochemistry to materials science, their aromatic flat surface is effective in the target binding, through π-stacking interactions, or to properly outdistance the substituents according to a specific orientation. Some of the recent designs of new oxadiazole based scaffolds include- oxalamine- a cough suppressant, Ataluren- used in treatment of duchenne muscular dystrophy and cystic fibrosis, Butalamine- vasodilator, Proxazole- for functional GI disorder and fasiplan- anxiolytic drug. Cancer being one of the deadliest diseases in the world accounts for millions of deaths every year. Widely used chemical and radiation therapies for cancer are not only ineffective in advanced stages of cancer, but also create additional side effects for patient. Currently, many anticancer drugs are available in the market that play an important role in cancer treatment, but concerns such as, drug resistance and side effects create an urgent need for the development of new anti-tumor drugs with high potency and less side effects. Hence, the aim of our study is to evaluate the inhibitory properties of new novel indole based diaza-sulphonamides against the JAK3 gene. The JAK3 gene provides instructions for making a protein that is critical for the normal development and function of the immune system. The JAK3 protein is part of a signaling pathway called the JAK/STAT pathway, which transmits chemical signals from outside the cell to the cell's nucleus. Signals relayed by the JAK3 protein regulate the growth and maturation of certain types of white blood cells called T cells, natural killer cells and B cells. Therefore, it is of interest to document the molecular docking analysis of Indole based diaza-sulphonamides with JAK-3 protein.

## Material and Methods:

## Protein preparation:

**Figure 1:**
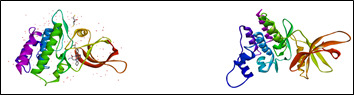
3D structure of human JAK-3 protein (PDB ID: 1YVJ).

The 3D crystal structure of the JAK-3 protein (PDB ID: 1YVJ) of *Homo sapiens* was downloaded from the protein data bank (Figure 1). As per standard protocol, protein preparation was done using the software Biovia Discovery Studio and Mgl tools 1.5.7. Water molecules and cofactors were chosen for elimination. The previously connected ligands were removed, and the protein was produced by adding polar hydrogens and Kollmans charges with Auto Prep.

## Ligand preparations:

**Figure 2:**
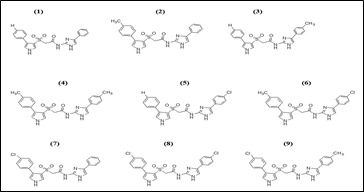
2D Structures of the indole based diaza-sulphonamide compounds (1-9)

The 2D structures of the literature derived indole based diaza-sulphonamide compounds (1-9) are drawn using the ChemDraw 16.0 software (Figure 2). During the optimization method, the software Chem3D was employed and all parameters were selected in order to achieve a stable structure with the least amount of energy. The structural optimization approach was used to estimate the global lowest energy of the title chemical. Each molecule's 3D coordinates (PDB) were determined using optimized structure.

## Auto dock Vina analysis:

**Figure 3:**
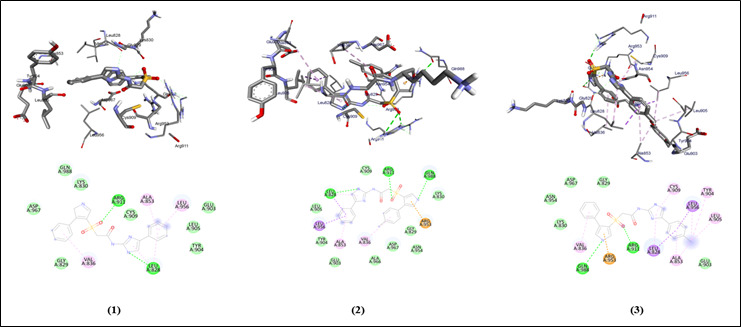
Molecular docking analysis of compounds (1-3) against the target JAK-3 protein of *Homo sapiens*

The graphical user interface Auto Dock vina was used for Ligand-Protein docking interactions (Figure 3). Auto Dock Tools (ADT), a free visual user interface (GUI) for the AutoDock Vina software, was used for the molecular docking research. The grid box was built with dimensions 39.4753, 27.4692, 33.2939 A pointing in the x, y, and z axes. The central grid box for 1YVJ was 1.4065, -11.5737, -13.9655 A. For each ligand, nine alternative conformations were created and ranked based on their binding energies utilizing Auto Dock Vina algorithms.

## Drug likeness and toxicity predictions:

In the present study, in-silico pharmacokinetic properties (ADME), drug-likeness, toxicity profiles are examined using SwissADME, and ProTox-II online servers. The SwissADME, a web tool from Swiss Institute of Bioinformatics (SIB) is used to convert the two-dimensional structures into their simplified molecular input line entry system (SMILES). The physicochemical properties (molar refractivity, topological polar surface area, number of hydrogen bond donors/ acceptors); pharmacokinetics properties (GI absorption, BBB permeation, P-gp substrate, cytochrome-P enzyme inhibition, skin permeation (log Kp)) which are critical parameters for prediction of the absorption and distribution of drugs within the body, and drug likeness (Lipinski's rule of five) were predicted using SwissADME. The toxicological endpoints (hepatotoxicity, carcinogenicity, immunotoxicity, mutagenicity) and the level of toxicity (LD50, mg/Kg) are determined using the ProTox-II server.

## Statistical analysis:

One way ANOVA was used for statistical analysis. The clinically proven drugs are used as a control and the results are compared. The significance of the results was found to be p< 0.05

## Results:

## Interaction of indole based diaza-sulphonamide compounds with JAK-3 protein from *Homo sapiens*:

All the compounds (1-9) are run against the target JAK-3 protein of *Homo sapiens* and it shows the range between -8.8 to -9.7 (Table 1). The compounds show hydrogen molecules interaction similar to clinically proven drug Doxorubicin (-9). All the compounds show similar binding affinity as the lead molecules are within the binding site.

## SwissADME and Lipinski’s rule of five:

The compounds show log Kp values between -6.3 to -6.54 cm/s (able 2). Compounds (4-9) show low gastro intestinal absorption so it needs a carrier molecule, whereas, compounds (1-3) show high gastro intestinal absorption so it doesn't need a carrier molecule. Compounds (1-9) show no blood brain barrier permeability. All the compounds (1-9) obey Lipinski's rule of five (Table 3).

## Toxicity profiling:

The compounds show class 4 toxicity (Table 4). All the compounds (1-9) show a similar LD50 value (720 mg/kg). Compounds (1-4) are inactive in hepatotoxicity, carcinogenicity, immunotoxicity, mutagenicity and cytotoxicity. However, compounds (5-9) are hepatotoxic.

## Discussion:

JAK-3, a cancer protein, also known as the JAK3 gene is a stable well-binding protein of the ginkgetin which has a well confirmed stereochemistry and is a high-quality structure with 90% residue in its most favored region. A leading drug that specifically suppresses JAK3 activity is NSC114792. Therefore, this JAK3 small molecule inhibitor may be a good place to start when creating a new class of medications that target JAK3 activity and may be useful in treating a variety of disorders that are brought on by aberrant JAK3 activity. After evaluation of the results, all the newly formed compounds showed high negative log kp value hence proving the fact that they are skin permeable. All the compounds can be absorbed in the gastrointestinal tract and none of them are blood-brain barrier permeable. The compounds (1-4) are non-toxic while compounds 5-9 are hepatotoxic. The compounds 1, 2, 3 and 4 are better in terms of toxicity than the clinically proven drugs. The selected ligands (1-4) show better interactions with module protein within the binding sites. Ligands 1, 2, 3 and 4 obey Lipinski's rule of five with no toxicity profile. Lipinski's rule of five is a rule of thumb that describes the drug ability of a determinate molecule. It states that there should be No more than 5 hydrogen bond donors. No more than 10 hydrogen bond acceptors. Molecular mass should not be less than 500Da and partition coefficient should not be greater than 5. The violation of 2 or more of these conditions predicts a molecule as a non- orally available drug. To sum it up, the compounds 1, 2, 3 and 4 have the potential to be used against cancer as they show better interaction than the clinical proven drugs, and are non-toxic. Furthermore, despite the fact that their mode of action needs to be defined, other oxadiazole ligands have been discovered with promise anticancer activity. It should be noted that oxadiazoles with significant cytotoxicity share a number of structural characteristics. In particular, the molecules described here have bioactive compound modifications, extended aromatic surfaces, or sulfur-based functional groups.

## Conclusion:

The selected compounds (1-4) show better interaction than the clinically proven drugs. Among the 9 compounds, 1, 2, 3 and 4 are potential to be lead molecules as they obey Lipinski's rule of five and are non-toxic. Therefore, these compounds are potential inhibitors for cancer protein JAK-3. It should be noted that further validation is required using in vitro studies.

## Figures and Tables

**Table 1:** Molecular docking interaction of indole based diaza-sulphonamide compounds (1-9) against JAK-3 protein of *Homo sapiens* (PDB ID: 1YVJ)

Ligands	Docking scores/Affinity (kcal/mol)	H-bond	Amino Acid Residual interactions	
			Hydrophobic/Pi-Cation	Van dar Waals
1	-9.3	Arg-911, Leu-828		Gly-829, Asp-967, Gln-988, Lys-830, Cys-909, Glu-903, Leu-905, Tyr-904
2	-9.7	Leu-828, Arg-911, Gln-988	Leu-956, Ala-853, Val-836, Arg-953	Cys-909, Lys-830, Gly-829, Asn-954, Asp-967, Ala-966, Glu-903, Tyr-904, Leu-905,
3	-9	Gln-988, Arg-911,	Val-836, Arg-953, Leu-828, Ala-853, Leu-905, Tyr-904, Leu-956, Cys-909	Glu-903, Lys-830, Asn-954, Asp-967, Gly-829
4	-9.8	Cys-909	Lys-855, Val-836, Gly-829, Leu-828, Ala-853, Leu-956, Asn-954	Leu-905, Asp-912, Asp-967, Phe-833, Gly-834, Ser-835, Gly-831, Lys-830
5	-8.8	Arg-911	Arg-953, Gln-988, Val-836, Ala-853, Val-884, Met-902, Leu-956, Leu-828	Asn-954, Lys-830, Gly-829, Leu-905, Glu-903,
6	-9.7	Arg-953, Cys-909	Lys-855, Asp-967, Val-836, Leu-828, Leu-956, Met-902, Ala-853, Gly-829, Asn-954	Gln-988, Phe-833, Gly-908, Tyr-904, Leu-905, Asp-912
7	-9.6	Arg-911, Gln-988	Leu-828, Leu-956, Ala-853, Val-836, Arg-953	Cys-909, Lys-830, Gly-829, Asn-954, Asp-967, Glu-903, Tyr-904, Leu-905
8	-9.6	Cys-909	Val-836, Leu-828, Leu-956, Met-902, Ala-853, Gly-829, Asp-967, Lys-855	Gln-988, Gly-908, Leu-905, Asn-954, Phe-833
9	-9.3	Arg-911	Val-836, Cys-909, Ala-853, Leu-905, Tyr-904, Leu-828, Leu-956, Arg-953	Cly-829, Gln-988, Lys-830, Glu-903
Doxorubicin	-9	Gln-988, Asp-967,	Leu-828, Val-836, Leu-956	Leu-905, Tyr-904, Val-884, Ala-966, Phe-968, Asn-954, Arg-953, Asp-912, Cys-909
Paclitaxel	-8	Cys-909, Arg-911, Arg-916,	Leu-956, Leu-828, Ala-853, Arg-953, Asp-912, Gly-908	Asn-954, Leu-905, Pro-906, Tyr-904
Tamoxifen	-7.5		Cys-909, Leu-828, Leu-956, Gly-829, Val-836	Asn-954, Gly-908, Leu-905, Asp-967, Lys-855, Ser-835, Gly-831, Gln-988

**Table 2:** SwissADME values of selected indole based diaza-sulphonamide compounds (1-9)

Compound	log Kp (cm/s)	GI absorption	BBB permeant	Pgp substrate	CYP1A2 inhibitor	CYP2C19 inhibitor	CYP2C9 inhibitor	CYP2D6 inhibitor	CYP3A4 inhibitor
1	-6.54	High	No	No	Yes	Yes	Yes	Yes	Yes
2	-6.36	High	No	No	Yes	Yes	Yes	Yes	Yes
3	-6.36	High	No	No	Yes	Yes	Yes	Yes	Yes
4	-6.19	Low	No	No	Yes	Yes	Yes	Yes	Yes
5	-6.3	Low	No	No	Yes	Yes	Yes	Yes	Yes
6	-6.12	Low	No	No	Yes	Yes	Yes	Yes	Yes
7	-6.3	Low	No	No	Yes	Yes	Yes	Yes	Yes
8	-6.06	Low	No	No	Yes	Yes	Yes	Yes	Yes
9	-6.12	Low	No	No	Yes	Yes	Yes	Yes	Yes
Doxorubicin	-8.71	Low	No	Yes	No	No	No	No	No
Paclitaxel	-8.91	Low	No	Yes	No	No	No	No	No
Tamoxifen	-3.5	Low	No	Yes	No	Yes	No	Yes	No

**Table 3:** Lipinski and Veber rules of selected indole based diaza-sulphonamide compounds (1-9)

Compound	MW	iLogP	HBD (nOHNH)	HBA (nON)	nrotb	MR	TPSA	Lipinski #violations	Bio availability score
1	406.46	1.21	3	4	7	110.74	116.09	0	0.55
2	420.48	1.14	3	4	7	115.7	116.09	0	0.55
3	420.48	1.6	3	4	7	115.7	116.09	0	0.55
4	434.51	1.66	3	4	7	120.67	116.09	0	0.55
5	440.9	1.54	3	4	7	115.75	116.09	0	0.55
6	454.93	1.7	3	4	7	120.71	116.09	0	0.55
7	440.9	1.68	3	4	7	115.75	116.09	0	0.55
8	475.35	1.92	3	4	7	120.76	116.09	0	0.55
9	454.93	1.9	3	4	7	120.71	116.09	0	0.55
Doxorubicin	543.52	2.16	6	12	5	132.66	206.07	3	0.17
Paclitaxel	853.91	4.51	4	14	15	218.96	221.29	2	0.17
Tamoxifen	371.51	4.64	0	2	8	119.72	12.47	1	0.55

**Table 4:** Toxicity profile of selected indole based diaza-sulphonamide compounds (1-9)

	TOXICITY						
Compound	^a^LD_50_ (mg/kg)	Class	HEPATOTOXICITY	CARCINOGENICITY	IMMUNOTOXICITY	MUTAGENICITY	CYTOTOXICITY
1	720	4	Inactive	Inactive	Inactive	Inactive	Inactive
2	720	4	Inactive	Inactive	Inactive	Inactive	Inactive
3	720	4	Inactive	Inactive	Inactive	Inactive	Inactive
4	720	4	Inactive	Inactive	Inactive	Inactive	Inactive
5	720	4	Active	Inactive	Inactive	Inactive	Inactive
6	720	4	Active	Inactive	Inactive	Inactive	Inactive
7	720	4	Active	Inactive	Inactive	Inactive	Inactive
8	720	4	Active	Inactive	Inactive	Inactive	Inactive
9	720	4	Active	Inactive	Inactive	Inactive	Inactive
Doxorubicin	205	3	Inactive	Inactive	Active	Active	Active
Paclitaxel	134	3	Inactive	Inactive	Active	Inactive	Active
Tamoxifen	1190	4	Active	Inactive	Active	Inactive	Inactive
^a^LD_50_: lethal dose parameter
